# A First-Principles Comparative Study on the Elastic and Related Properties of Ti_3_AC_2_ (A = Si, Ir, and Au) MAX Phases

**DOI:** 10.3390/ma18102296

**Published:** 2025-05-15

**Authors:** Yufeng Wen, Huaizhang Gu, Yanlin Yu, Zhangli Lai, Xianshi Zeng, Guilian Wang

**Affiliations:** 1 School of Science, Kaili University, Kaili 556011, China; kluwenyf@163.com (Y.W.); guhzh666@163.com (H.G.); 2 School of Mathematical Sciences and Physics, Jinggangshan University, Ji’an 343009, China; 15979627768@163.com (Z.L.); zengxueliang@163.com (X.Z.); 3 School of Electronic and Electrical Engineering, Shanghai University of Engineering Science, Shanghai 201620, China; wglwrc2016@126.com

**Keywords:** MAX phases, elastic properties, mechanical properties, acoustic properties, thermal properties, first principles

## Abstract

The elastic, mechanical, acoustic, and thermal properties of ${\mathrm{Ti}}_{3}$Si$C_{2}$, ${\mathrm{Ti}}_{3}$Ir$C_{2}$, and ${\mathrm{Ti}}_{3}$Au$C_{2}$ MAX phases were systematically investigated using first-principles calculations based on density functional theory. The computed lattice parameters and elastic, mechanical, and acoustic properties were consistent with existing experimental and theoretical findings, confirming the intrinsic mechanical stability of these MAX phases. Single-crystal elastic stiffness constants were used to derive polycrystalline elastic moduli, directional dependencies of bulk, shear, and Young’s moduli, and anisotropic factors. The results revealed a ductility sequence of ${\mathrm{Ti}}_{3}$Si$C_{2}$ < ${\mathrm{Ti}}_{3}$Ir$C_{2}$ < ${\mathrm{Ti}}_{3}$Au$C_{2}$, with ${\mathrm{Ti}}_{3}$Ir$C_{2}$ and ${\mathrm{Ti}}_{3}$Au$C_{2}$ exhibiting greater elastic anisotropy than ${\mathrm{Ti}}_{3}$Si$C_{2}$. Additionally, sound velocities, Debye temperatures, minimum thermal conductivities, melting points, and Grüneisen parameters were determined. The findings showed that ${\mathrm{Ti}}_{3}$Si$C_{2}$ outperforms ${\mathrm{Ti}}_{3}$Ir$C_{2}$ and ${\mathrm{Ti}}_{3}$Au$C_{2}$ in sound velocity, average sound velocity, Debye temperature, and minimum thermal conductivity, while ${\mathrm{Ti}}_{3}$Ir$C_{2}$ has the highest melting point and ${\mathrm{Ti}}_{3}$Au$C_{2}$ the largest Grüneisen parameter. These results provide valuable insights into the design of related materials for high-performance applications.

## 1. Introduction

MAX phases represent an extensive family of ternary nanolaminated compounds with the chemical formula of $M_{n+1}$A$X_{n}$ (*n* = 1, 2, or 3), where “M” denotes an early transition metal, “A” represents an A-group element typically from groups IIIA or IVA of the periodic table, and “X” stands for C and or N. Due to their unique layered structure, comprising alternating metallic and X layers, and their diverse bonding features, stemming from strong covalent-ionic and weak metallic bonds, MAX phases display a hybrid nature, integrating metallic and ceramic properties. This includes high temperature stability, remarkable mechanical properties, high thermal conductivity, and excellent resistance to oxidation [1,2,3]. The exceptional mechanical and thermal properties of MAX phases make them ideal candidates for application in harsh environments [3]. Elastic stiffness constants are fundamental measures that describe how solid materials respond to external mechanical forces. Understanding these constants is crucial for various practical applications that rely on the mechanical behavior of solids. The elastic properties of solids are closely tied to fundamental solid-state phenomena, such as interatomic bonding, equations of state, and phonon spectra. They are also thermodynamically connected to specific heat, thermal expansion, Debye temperature, and the Grüneisen parameter. Recently, Fashandi et al. [4] synthesized novel ${\mathrm{Ti}}_{3}$Au$C_{2}$ and ${\mathrm{Ti}}_{3}$${\mathrm{Au}}_{2}$$C_{2}$ phases via a substitutional solid-state reaction of Au into ${\mathrm{Ti}}_{3}$Si$C_{2}$ single-crystal thin films, accompanied by Si out-diffusion. They further produced ${\mathrm{Ti}}_{3}$Ir$C_{2}$ phase by substituting Ir for Au in ${\mathrm{Ti}}_{3}$${\mathrm{Au}}_{2}$$C_{2}$. In order to explore the possibility of application in harsh environments, it is necessary to study the mechanical and thermal properties of these novel phases. To the best of our knowledge, the elastic, mechanical, acoustic, and thermal properties of these novel phases have not yet been experimentally investigated, despite the fact that those of the ${\mathrm{Ti}}_{3}$Si$C_{2}$ MAX phase have been determined using pulse-echo ultrasonic techniques [5] and resonant ultrasound spectroscopy [6]. These motivate us to comparatively investigate the elastic, mechanical, acoustic, and thermal properties of ${\mathrm{Ti}}_{3}$Si$C_{2}$, ${\mathrm{Ti}}_{3}$Ir$C_{2}$, and ${\mathrm{Ti}}_{3}$Au$C_{2}$ MAX phases.

First-principles calculations are among the most effective methods for exploring material properties. Over the past decades, first-principles calculations have been widely employed to investigate the properties of MAX phases [1,4,7,8,9,10,11,12,13,14,15,16]. Among the three MAX phases of ${\mathrm{Ti}}_{3}$Si$C_{2}$, ${\mathrm{Ti}}_{3}$Au$C_{2}$, and ${\mathrm{Ti}}_{3}$Ir$C_{2}$, the various properties of ${\mathrm{Ti}}_{3}$Si$C_{2}$, which include phase stability, electronic, elastic, mechanical, thermal, optical, and other properties, have been investigated by some researchers using first-principles calculations [4,7,8,9,10,11]. For ${\mathrm{Ti}}_{3}$Au$C_{2}$, the phase stability, electronic, elastic, mechanical, thermal, and optical properties have also been investigated by Fashandi et al. [4] and Qureshi et al. [12] using first-principles calculations. For ${\mathrm{Ti}}_{3}$Ir$C_{2}$, only the phase stability and electronic structures have been studied by Fashandi et al. [4] using first-principles calculations. To date, the anisotropy in the acoustic properties of ${\mathrm{Ti}}_{3}$Si$C_{2}$ and ${\mathrm{Ti}}_{3}$Au$C_{2}$ has not been theoretically investigated, nor have the elastic, mechanical, acoustic, and thermal properties of ${\mathrm{Ti}}_{3}$Ir$C_{2}$ been thoroughly explored. Meanwhile, no systematical study has been conducted on the properties of ${\mathrm{Ti}}_{3}$Si$C_{2}$, ${\mathrm{Ti}}_{3}$Ir$C_{2}$, and ${\mathrm{Ti}}_{3}$Au$C_{2}$ MAX phases. Accordingly, this work employs first-principles to comparatively investigate the elastic, mechanical, acoustic, and thermal properties of these MAX phases.

## 2. Methodology

**Figure 1:**
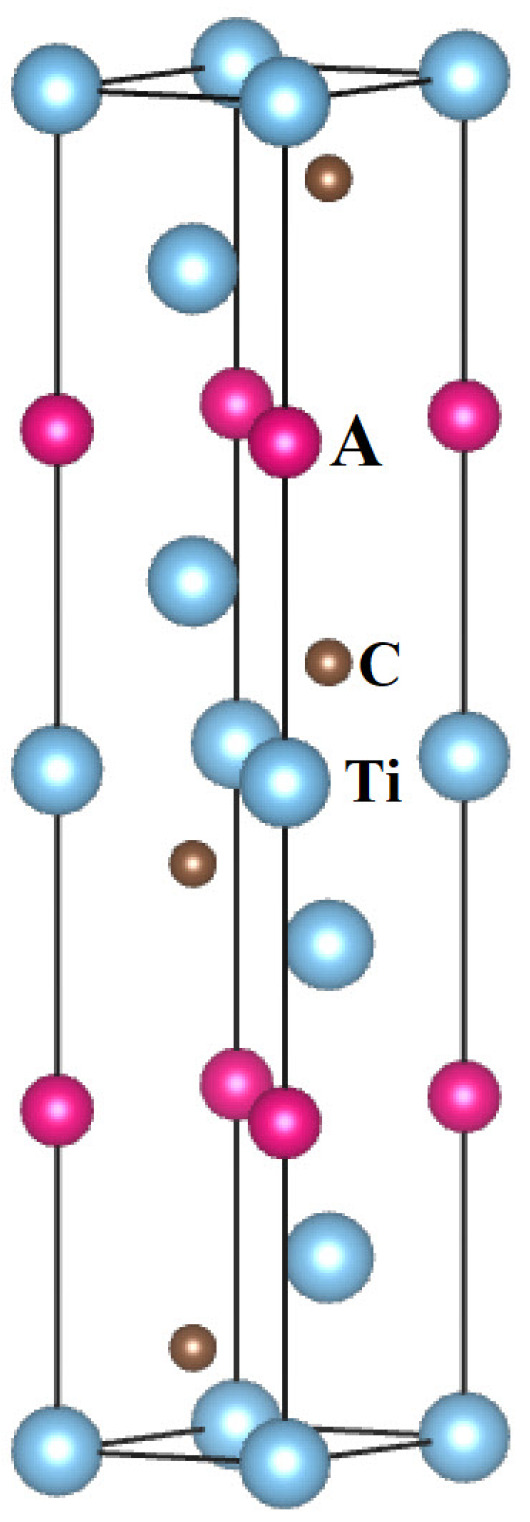
Crystal structure of ${\mathrm{Ti}}_{3}$A$C_{2}$ (A = Si, Au, and Ir) MAX phases.

The MAX phases of ${\mathrm{Ti}}_{3}$Si$C_{2}$, ${\mathrm{Ti}}_{3}$Au$C_{2}$, and ${\mathrm{Ti}}_{3}$Ir$C_{2}$ exhibit hexagonal symmetry with the space group P$6_{3}$/mmc. The atomic positions are as follows: Ti at 2*a* and 4*f*, A (Si, Au or Ir) at 2*b*, and C at 4*f* Wyckoff positions, as shown in Figure 1. The initial calculation models for these MAX phases were constructed using their theoretical lattice parameters and atomic positions as reported in Ref. [4]. For a hexagonal crystal, there are five independent single-crystal elastic stiffness constants: $C_{11}$, $C_{12}$, $C_{13}$, $C_{33}$, and $C_{44}$. The stress–strain method is an effective approach for determining these single-crystal constants, where the elastic stiffness constants are defined as the first derivatives of the stresses with respect to the strain tensor [17]. After performing first-principles calculations with full optimization of the lattice parameters and atomic coordinates of atoms in the unit cell, the elastic stiffness constants of these MAX phases were predicted using the strain–stress method based on first-principles calculations.

Ab initio computations were performed using density functional theory (DFT) with the projector-augmented wave technique [18], employing the Perdew–Burke–Ernzerhof (PBE) generalized gradient approximation (GGA) [19], as implemented in the Vienna Ab initio Simulation Package (VASP) [20,21,22]. The electronic wave functions were expanded with an energy cutoff of 520 eV, and the total energy convergence threshold was set to $10^{-6}$ eV during the self-consistency process. The Brillouin zone was sampled using Γ-centered Monkhorst-Pack k-point grids [23]. For structural optimization, a 15 × 15 × 2 k-point grid was utilized, while a denser 21 × 21 × 3 grid was employed for calculating elastic stiffness constants. The structure was relaxed by optimizing the volume, shape, and atomic positions until the residual forces on each atom were below 0.001 eV/Å. Elastic constants were determined by applying six finite lattice distortions and analyzing the strain–stress response within VASP. Both rigid-ion and relaxed-ion contributions to the elastic tensor were considered. The ionic contributions were computed by inverting the ionic Hessian matrix and incorporating the internal strain tensor [24,25]. The final elastic constants represent the combined effects of rigid-ion distortions and ionic relaxations, providing a comprehensive description of the material’s elastic behavior.

## 3. Results and Discussions

### 3.1. Elastic and Mechanical Properties

The calculated equilibrium lattice constants (*a*, *c*), mass density (ρ), single-crystal elastic stiffness constants ($C_{ij}$), and Cauchy pressures (${\mathrm{CP}}_{1}$, ${\mathrm{CP}}_{2}$) for ${\mathrm{Ti}}_{3}$Si$C_{2}$, ${\mathrm{Ti}}_{3}$Ir$C_{2}$, and ${\mathrm{Ti}}_{3}$Au$C_{2}$ are listed in Table 1 alongside existing theoretical data and experimental measurements. The optimized lattice constants of these MAX phases are in excellent agreement with previous calculations and experimental data [4,7,9,11,26,27]. The differences between our calculations and previous reports for the lattice constants *a* and *c* are less than 0.63%. For ${\mathrm{Ti}}_{3}$Si$C_{2}$, the deviations in the density compared to previous theoretical results [7] and experimental values [5,6] are 1.45% and 0.33%, respectively. Additionally, its elastic stiffness constants are consistent with previous theoretical results [7,8,9,10,12]. These findings demonstrate the accuracy and reliability of our calculations.

The intrinsic mechanical stability of a crystal usually can be determined using the elastic stiffness constants based on Born–Huang’s lattice dynamical theory [28]. According to Born–Huang’s theory, the mechanical stability criteria for hexagonal crystals are as follows:

(1) $$\left\{\begin{array}{c} C_{ii}>0,\;i=1,3,4,\\ \;C_{11}-C_{12}>0,\\ \left(C_{11}+C_{12}\right)C_{33}-2C_{13}^{2}>0. \end{array}\right.$$

The elastic stiffness constants of the three MAX phases satisfy the mechanical stability criteria, demonstrating their intrinsic stability. The constants $C_{11}$ and $C_{33}$ represent the rigidity against unidirectional deformation along the a and c axes, respectively. The values of $C_{11}$ and $C_{33}$ exceed those of other elastic stiffness constants, indicating incompressibility under uniaxial stress along the *a* and *c* axes. The value of $C_{11}$ for ${\mathrm{Ti}}_{3}$Si$C_{2}$ is slightly larger than $C_{33}$, implying stronger incompressibility along the *a* axis compared to the *c* axis. Conversely, ${\mathrm{Ti}}_{3}$Ir$C_{2}$ and ${\mathrm{Ti}}_{3}$Au$C_{2}$ exhibit slightly smaller $C_{11}$ values, indicating stronger compressibility along the *a* axis. For ${\mathrm{Ti}}_{3}$Si$C_{2}$ and ${\mathrm{Ti}}_{3}$Ir$C_{2}$, The compression modulus $C_{12}$ is smaller than $C_{13}$, indicating a weaker elastic modulus under bi-axial stress along the *a* axis compared to stress deviating from the a axis. Conversely, ${\mathrm{Ti}}_{3}$Au$C_{2}$ exhibits a larger $C_{12}$ value, indicating a stronger elastic modulus under bi-axial stress along the *a* axis compared to stress deviating from the *a* axis. The shear moduli ($C_{44}$ and $C_{66}$) exhibit deviations, indicating shear modulus anisotropy. Meanwhile, the $C_{11}$ and $C_{33}$ values of ${\mathrm{Ti}}_{3}$Si$C_{2}$ fall between those of ${\mathrm{Ti}}_{3}$Ir$C_{2}$ and ${\mathrm{Ti}}_{3}$Au$C_{2}$, indicating the incompressibility sequence along the principal axes is ${\mathrm{Ti}}_{3}$Ir$C_{2}$ > ${\mathrm{Ti}}_{3}$Si$C_{2}$ > ${\mathrm{Ti}}_{3}$Au$C_{2}$.

Pettifor et al. [29] suggested that the angular nature of atomic bonding in materials, affecting whether a material is brittle or ductile, can be explained using the Cauchy pressure. They showed that brittleness stems from an unusual characteristic of elastic constants, specifically a negative Cauchy pressure. Metallic bonding generally exhibits positive Cauchy pressure, whereas directional bonding shows negative values, with more negative values signifying stronger directional bonding characteristics. For hexagonal crystals, the Cauchy pressures are given by ${\mathrm{CP}}_{1}=C_{13}-C_{44}$ and ${\mathrm{CP}}_{2}=C_{12}-C_{66}$. The corresponding values for the three MAX phases are listed in Table 1. ${\mathrm{Ti}}_{3}$Si$C_{2}$ exhibits negative Cauchy pressures, indicating brittle bonding with directional characteristics. In contrast, ${\mathrm{Ti}}_{3}$Au$C_{2}$ demonstrates metallic bonding and ductility, as evidenced by positive Cauchy pressures. For ${\mathrm{Ti}}_{3}$Ir$C_{2}$, ${\mathrm{CP}}_{1}$ is positive, while ${\mathrm{CP}}_{2}$ is negative, suggesting a mix of metallic and directional bonding. Moreover, ${\mathrm{CP}}_{1}$ exceeding twice the absolute value of ${\mathrm{CP}}_{2}$ indicates that metallic bonding is dominant, contributing to the ductility of ${\mathrm{Ti}}_{3}$Ir$C_{2}$.

Bulk modulus (*B*) represents the material’s resistance to hydrostatic pressure. Shear modulus (*G*) characterizes a material’s resistance to shear deformation. Young’s modulus (*E*) quantifies the resistance to uniaxial tension or compression. Using the Voigt–Reuss–Hill averaging scheme [30], the polycrystalline bulk, shear, Young’s moduli, and Poisson’s ratio (σ) of ${\mathrm{Ti}}_{3}$Si$C_{2}$, ${\mathrm{Ti}}_{3}$Au$C_{2}$, and ${\mathrm{Ti}}_{3}$Ir$C_{2}$ can be determined from the obtained single-crystal elastic stiffness constants $C_{ij}$. For hexagonal structures, the Voigt ($B_{V}$) and Reuss ($B_{R}$) bulk moduli and Voigt ($G_{V}$) and Reuss ($G_{R}$) shear moduli are expressed as follows [31,32]:

(2) $$\left\{\begin{array}{c} B_{V}=\left[2\left(C_{11}+C_{12}\right)+4C_{13}+C_{33}\right]/9,\\ \;G_{V}=\left[M+12\left(C_{44}+C_{66}\right)\right]/30,\\ \;B_{R}=C/M,\\ \;G_{R}=5CC_{44}C_{66}/\left[6B_{V}C_{44}C_{66}+2C\left(C_{44}+C_{66}\right)\right],\\ \;M=C_{11}+C_{12}+2C_{33}-4C_{13},\\ \;C=\left(C_{11}+C_{12}\right)C_{33}-2C_{13}^{2}. \end{array}\right.$$

According to the averaging scheme, the bulk and shear moduli are estimated as follows:

(3) $$\left\{\begin{array}{c} B_{H}=\left(B_{V}+B_{R}\right)/2,\\ \;G_{H}=\left(G_{V}+G_{R}\right)/2. \end{array}\right.$$

Young’s modulus ($E_{H}$) and Poisson’s ratio ($\sigma_{H}$) can be further obtained by the following expressions:

(4) $$\left\{\begin{array}{c} E_{H}=9B_{H}G_{H}/\left(3B_{H}+G_{H}\right)\\ \;\sigma_{H}=\left(3B_{H}-2G_{H}\right)/\left(6B_{H}+2G_{H}\right) \end{array}\right.$$

The calculated elastic moduli *B*, *G*, *E*, Poisson’s ratio σ, and G/B ratio for polycrystalline ${\mathrm{Ti}}_{3}$Si$C_{2}$, ${\mathrm{Ti}}_{3}$Ir$C_{2}$, and ${\mathrm{Ti}}_{3}$Au$C_{2}$ are listed in Table 2, along with theoretical values and experimental data. The values of *B*, *G*, *E*, σ, and the G/B ratio agree well with those of previous calculations [7,8,9,10,11] and experimental measurements [5,6]. For these isostructural 312 phases, the bulk modulus *B* follows the sequence ${\mathrm{Ti}}_{3}$Ir$C_{2}$ > ${\mathrm{Ti}}_{3}$Si$C_{2}$ > ${\mathrm{Ti}}_{3}$Au$C_{2}$. Conversely, both the shear modulus *G* and Young’s modulus *E* decrease in the order ${\mathrm{Ti}}_{3}$Si$C_{2}$ > ${\mathrm{Ti}}_{3}$Ir$C_{2}$ > ${\mathrm{Ti}}_{3}$Au$C_{2}$. Generally, a higher shear modulus *G* indicates stronger directional bonding between atoms. Our calculations reveal that ${\mathrm{Ti}}_{3}$Si$C_{2}$ exhibits the strongest bonding, followed by ${\mathrm{Ti}}_{3}$Ir$C_{2}$, while ${\mathrm{Ti}}_{3}$Au$C_{2}$ has the weakest bonding. which is consistent with our calculation of the Cauchy pressures. Through empirical observations, Pugh [33] established a direct relationship between the plastic behavior of materials and their elastic properties. A greater G/B ratio is commonly linked to brittle characteristics, while a lesser ratio is indicative of ductility. The dividing line between ductile and brittle materials is often cited as 0.5; materials with G/B above this threshold are typically brittle, whereas those below are more ductile. In our case, the calculated results clearly imply that ${\mathrm{Ti}}_{3}$Si$C_{2}$ is a brittle phase, whereas both ${\mathrm{Ti}}_{3}$Ir$C_{2}$ and ${\mathrm{Ti}}_{3}$Au$C_{2}$ are ductile phases. Meanwhile, the ductility is increased in the sequence of ${\mathrm{Ti}}_{3}$Si$C_{2}$ < ${\mathrm{Ti}}_{3}$Ir$C_{2}$ < ${\mathrm{Ti}}_{3}$Au$C_{2}$. Poisson’s ratio σ is the most informative regarding the bonding behavior among all elastic moduli [34]. The typical value of σ is 0.1, 0.25, and 0.33 for covalent, ionic, and metallic materials, respectively [35]. The present results of Poisson’s ration indicate that ${\mathrm{Ti}}_{3}$Si$C_{2}$ exhibits ionic/covalent bonding characteristics, while ${\mathrm{Ti}}_{3}$Ir$C_{2}$ and ${\mathrm{Ti}}_{3}$Au$C_{2}$ distinctly belong to the class of metallically bonded materials. Meanwhile, the value of σ for ${\mathrm{Ti}}_{3}$Ir$C_{2}$ is slightly smaller than that of ${\mathrm{Ti}}_{3}$Au$C_{2}$, implying the stronger metallic bonding in ${\mathrm{Ti}}_{3}$Au$C_{2}$. This is also consistent with our calculation of the Cauchy pressures.

Elastic anisotropy plays a crucial role in assessing the mechanical properties of materials. A three-dimensional (3D) surface model is used to depict elastic anisotropy by showing how the elastic modulus changes with crystallographic orientation [36]. The directional dependence of *B*, *G*, and *E* in the hexagonal crystal system can be described using the equations below:

(5) $$\left\{\begin{array}{c} B^{-1}=\left(S_{11}+S_{12}+S_{13}\right)-\left(S_{11}+S_{12}-S_{13}-S_{33}\right)l_{3}^{2},\\ \;E^{-1}=S_{11}{\left(l_{1}^{2}+l_{2}^{2}\right)}^{2}+S_{33}l_{3}^{4}+\left(2S_{13}+S_{44}\right)\left(l_{1}^{2}l_{3}^{2}+l_{2}^{2}l_{3}^{2}\right),\\ \;G^{-1}=4S_{11}\left(l_{1}^{2}m_{1}^{2}+l_{2}^{2}m_{2}^{2}\right)+4S_{33}l_{3}^{2}m_{3}^{2}+8S_{12}l_{1}m_{1}l_{2}m_{2}+8S_{13}\left(l_{2}m_{2}+l_{1}m_{1}\right)l_{3}m_{3}\\ \;+S_{44}\left[{\left(l_{2}m_{3}+m_{2}l_{3}\right)}^{2}+{\left(l_{1}m_{3}+m_{1}l_{3}\right)}^{2}\right]+S_{66}{\left(l_{1}m_{2}+m_{1}l_{2}\right)}^{2}. \end{array}\right.$$

**Figure 2:**
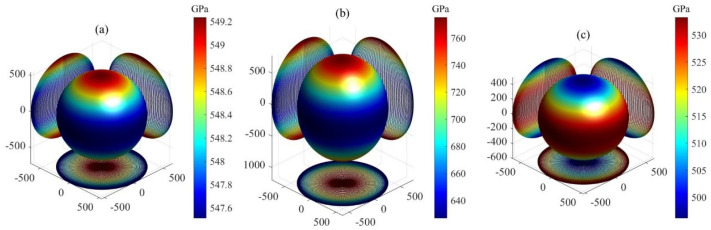
The orientation dependence of bulk modulus for MAX phases: (**a**) ${\mathrm{Ti}}_{3}$Si$C_{2}$, (**b**) ${\mathrm{Ti}}_{3}$Ir$C_{2}$, and (**c**) ${\mathrm{Ti}}_{3}$Au$C_{2}$.

**Figure 3:**
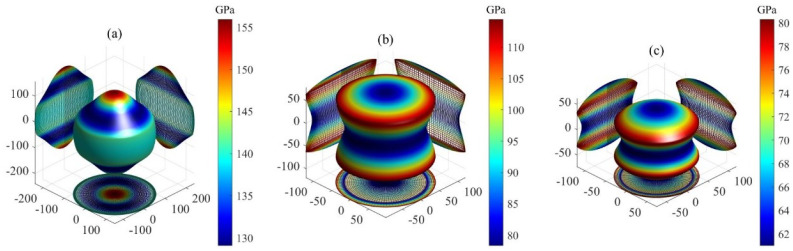
The orientation dependence of shear modulus for MAX phases: (**a**) ${\mathrm{Ti}}_{3}$Si$C_{2}$, (**b**) ${\mathrm{Ti}}_{3}$Ir$C_{2}$ and (**c**) ${\mathrm{Ti}}_{3}$Au$C_{2}$.

**Figure 4:**
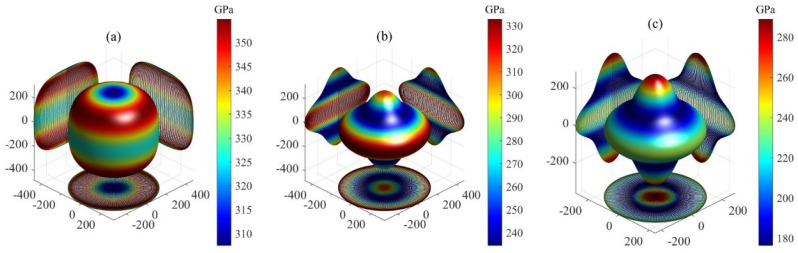
The orientation dependence of Young’s modulus for MAX phases: (**a**) ${\mathrm{Ti}}_{3}$Si$C_{2}$, (**b**) ${\mathrm{Ti}}_{3}$Ir$C_{2}$, and (**c**) ${\mathrm{Ti}}_{3}$Au$C_{2}$.

Here, the elastic compliance constants $S_{ij}$ constitute the inverse matrix of elastic stiffness constants $C_{ij}$, while the direction cosines are defined as $l_{1}=l_{11}$, $l_{2}=l_{12}$, $l_{3}=l_{13}$, $m_{1}=l_{21}$, $m_{2}=l_{22}$, and $m_{3}=l_{23}$. The 3D plots showing the orientation dependence of *B*, *G*, and *E* for ${\mathrm{Ti}}_{3}$Si$C_{2}$, ${\mathrm{Ti}}_{3}$Ir$C_{2}$, and ${\mathrm{Ti}}_{3}$Au$C_{2}$ are presented in Figure 2, Figure 3 and Figure 4, respectively. All the plots are axis-symmetrical around the *c* axis. This axis symmetry arises from the hexagonal symmetry. For instance, considering the bulk modulus, where $l_{1}^{2}+l_{2}^{2}+l_{3}^{2}=1$, a specific value of $l_{3}$ dictates $l_{1}^{2}+l_{2}^{2}$, thereby determining B and resulting in the 3D plot’s axis symmetry around the *c* axis. For isotropic materials, the 3D curved surface is spherical. However, real crystals, being anisotropic, exhibit distorted spheres, with the degree of distortion reflecting their anisotropic nature. All 3D plots exhibit nonspherical shapes, indicating varying degrees of elastic anisotropy in these three MAX phases. For bulk and shear moduli, the deviation from sphericity decreases in the order ${\mathrm{Ti}}_{3}$Ir$C_{2}$ > ${\mathrm{Ti}}_{3}$Au$C_{2}$ > ${\mathrm{Ti}}_{3}$Si$C_{2}$. The degree of nonsphericity for Young’s modulus increases in the order ${\mathrm{Ti}}_{3}$Si$C_{2}$ < ${\mathrm{Ti}}_{3}$Ir$C_{2}$ < ${\mathrm{Ti}}_{3}$Au$C_{2}$. Notably, the shear and Young’s moduli exhibit significantly nonspherical shapes compared to the corresponding bulk moduli, indicating a higher degree of anisotropy in shear and Young’s moduli compared to the bulk modulus.

The uniaxial bulk moduli along the *a*-axis ($B_{a}$) and *c*-axis ($B_{C}$) are defined as [37]:

(6) $$\left\{\begin{array}{c} B_{a}=1/\left(S_{11}+S_{12}+S_{13}\right),\\ \;B_{C}=1/\left(2S_{13}+S_{33}\right). \end{array}\right.$$

The average shear modulus *G*, Young’s modulus *E*, and Poisson’s ratio σ on the (2$\bar{1}$0), (010), and (001) planes can be determined using the following relationships [37]:

(7) $$\left\{\begin{array}{c} G_{1}=G\left(2\bar{1}0\right)=G\left(010\right)=2/\left(S_{44}+2S_{11}-2S_{12}\right),\\ \;G_{2}=G\left(001\right)=1/S_{44}. \end{array}\right.$$

(8) $$\left\{\begin{array}{c} E_{1}=E\left(2\bar{1}0\right)=E\left(010\right)=1/S_{11},\\ \;E_{2}=E\left(001\right)=1/S_{33}. \end{array}\right.$$

(9) $$\left\{\begin{array}{c} \sigma_{1}=\sigma \left(2\bar{1}0\right)=\sigma \left(010\right)=-\left(S_{12}+S_{13}\right)/2S_{11},\\ \;\sigma_{2}=\sigma \left(001\right)=-S_{13}/S_{33}. \end{array}\right.$$

The calculated values of the uniaxial modulus *B*, shear modulus *G*, Young’s modulus *E*, and Poisson’s ratio σ on the (2$\bar{1}$0), (010), and (001) planes are presented in Table 3. The $E_{1}$, $E_{2}$, $\sigma_{1}$, and $\sigma_{2}$ values of ${\mathrm{Ti}}_{3}$Si$C_{2}$ obtained in this study are in excellent agreement with those of previous calculations [11]. For ${\mathrm{Ti}}_{3}$Si$C_{2}$ and ${\mathrm{Ti}}_{3}$Ir$C_{2}$, $B_{a}$ is smaller than $B_{C}$, whereas for ${\mathrm{Ti}}_{3}$Au$C_{2}$, $B_{a}$ is larger than $B_{C}$. For the average shear modulus, $G_{1}<G_{2}$ for ${\mathrm{Ti}}_{3}$Si$C_{2}$, whereas $G_{1}>G_{2}$ for ${\mathrm{Ti}}_{3}$Ir$C_{2}$ and ${\mathrm{Ti}}_{3}$Au$C_{2}$. For ${\mathrm{Ti}}_{3}$Si$C_{2}$ and ${\mathrm{Ti}}_{3}$Ir$C_{2}$, $E_{1}$ is larger than $E_{2}$, whereas for ${\mathrm{Ti}}_{3}$Au$C_{2}$, $E_{1}$ is smaller than $E_{2}$. Similar to the uniaxial bulk modulus, $\sigma_{1}$ is smaller than $\sigma_{2}$ for ${\mathrm{Ti}}_{3}$Si$C_{2}$ and ${\mathrm{Ti}}_{3}$Ir$C_{2}$, whereas for ${\mathrm{Ti}}_{3}$Au$C_{2}$, $\sigma_{1}$ is larger than $\sigma_{2}$. These results demonstrate that the anisotropic behaviors of ${\mathrm{Ti}}_{3}$Si$C_{2}$, ${\mathrm{Ti}}_{3}$Ir$C_{2}$, and ${\mathrm{Ti}}_{3}$Au$C_{2}$ are significant due to the differences in shear modulus, Young’s modulus, and Poisson’s ratio between the prismatic planes (2$\bar{1}$0), (010), and the basal plane (001). Additionally, both $B_{a}$ and $B_{C}$ decrease in the order ${\mathrm{Ti}}_{3}$Ir$C_{2}$ > ${\mathrm{Ti}}_{3}$Si$C_{2}$ > ${\mathrm{Ti}}_{3}$Au$C_{2}$, indicating that the incompressibility along the a and c axes follows the same sequence. This is consistent with the elastic constants $C_{11}$ and $C_{33}$ of these MAX phases. For ${\mathrm{Ti}}_{3}$Si$C_{2}$, both $G_{1}$ and $G_{2}$ are the largest, followed by ${\mathrm{Ti}}_{3}$Ir$C_{2}$, with ${\mathrm{Ti}}_{3}$Au$C_{2}$ having the smallest values. Similarly, $E_{1}$ and $E_{2}$ decrease in the order ${\mathrm{Ti}}_{3}$Ir$C_{2}$ > ${\mathrm{Ti}}_{3}$Si$C_{2}$ > ${\mathrm{Ti}}_{3}$Au$C_{2}$, consistent with $B_{a}$ and $B_{C}$. For Poisson’s ratio, $\sigma_{1}$ increases in the order ${\mathrm{Ti}}_{3}$Si$C_{2}$ < ${\mathrm{Ti}}_{3}$Ir$C_{2}$ < ${\mathrm{Ti}}_{3}$Au$C_{2}$, while $\sigma_{2}$ for ${\mathrm{Ti}}_{3}$Si$C_{2}$ lies between those for ${\mathrm{Ti}}_{3}$Ir$C_{2}$ and ${\mathrm{Ti}}_{3}$Au$C_{2}$.

Another approach to investigate elastic anisotropy involves various anisotropic factors. The shear anisotropy factor quantifies the degree of anisotropy based on atomic bonding in different planes. For hexagonal crystals, it is defined as follows [37]:

(10) $$A_{1}=\frac{4C_{44}}{\left(C_{11}+C_{33}-2C_{13}\right)},$$

for the {100} shear planes between <011> and <010> directions,

(11) $$A_{2}=\frac{4C_{55}}{\left(C_{22}+C_{33}-2C_{23}\right)},$$

for the {010} shear planes between <101> and <001> directions, and

(12) $$A_{3}=\frac{4C_{66}}{\left(C_{11}+C_{22}-2C_{12}\right)},$$

for the {001} shear planes between <110> and <010> directions. When $A_{1}$, $A_{2}$, and $A_{3}$ equal 1, the crystal is isotropic. Values deviating from 1 indicate varying degrees of anisotropic behavior. The calculated values of $A_{1}$, $A_{2}$, and $A_{3}$ are presented in Table 4. The $A_{1}$ and $A_{2}$ values for ${\mathrm{Ti}}_{3}$Si$C_{2}$, ${\mathrm{Ti}}_{3}$Au$C_{2}$, and ${\mathrm{Ti}}_{3}$Ir$C_{2}$ significant deviate from 1, indicating anisotropic behavior in {100} and {010} planes. Conversely, their $A_{3}$ values equal 1, indicating isotropy in {001} planes. These results are consistent with the planar projections of shear modulus in Figure 3, specifically for the (100), (010), and (001) planes. Ranganathan and Ostoja-Starzewski [38] introduced the universal anisotropy index ($A^{U}$) to quantify single-crystal elastic anisotropy. The universal anisotropy index $A^{U}$ is defined as follows:

(13) $$A^{U}=5\frac{G_{V}}{G_{R}}+\frac{B_{V}}{B_{R}}-6.$$

$A^{U}=0$ represents isotropic single crystals, while $A^{U}>0$ denotes the extent of anisotropy. The calculated $A^{U}$ values are 0.032 for ${\mathrm{Ti}}_{3}$Si$C_{2}$, 0.397 for ${\mathrm{Ti}}_{3}$Ir$C_{2}$, and 0.337 for ${\mathrm{Ti}}_{3}$Au$C_{2}$, indicating anisotropic behavior in these MAX phases. Kube [39] proposed the log-Euclidean anisotropy index ($A^{L}$) to quantify single-crystal elastic anisotropy. The log-Euclidean anisotropy index $A^{L}$ is defined as follows:

(14) $$A^{L}=\sqrt{{\left[\ln\left(\frac{B_{V}}{B_{R}}\right)\right]}^{2}+{5\left[\ln\left(\frac{G_{V}}{G_{R}}\right)\right]}^{2}}.$$

$A^{L}=0$ represents isotropic single crystals, while $A^{L}>0$ denotes the extent of anisotropy. The calculated $A^{L}$ values are 0.014 for ${\mathrm{Ti}}_{3}$Si$C_{2}$, 0.169 for ${\mathrm{Ti}}_{3}$Ir$C_{2}$, and 0.146 for ${\mathrm{Ti}}_{3}$Au$C_{2}$, indicating anisotropic elasticity in these phases. These results are consistent with the 3D surface representations earlier.

### 3.2. Acoustic and Thermal Properties

By utilizing the elastic constants of single crystals, the acoustic wave velocities corresponding to longitudinal and transverse wave modes can be determined through Bragger’s method. The velocities of hexagonal crystals in their principal orientations can be readily determined by the following [37]:

(15) $$\left\{\begin{array}{c} \left[100\right]v_{l}=\left[010\right]v_{l}=\sqrt{(C_{11}-C_{12})/2\rho }\\ \;\left[010\right]v_{t1}=\sqrt{C_{11}/\rho }\\ \;\left[001\right]v_{t2}=\sqrt{C_{44}/\rho }, \end{array}\right.$$

(16) $$\left\{\begin{array}{c} \left[001\right]v_{l}=\sqrt{C_{33}/\rho }\\ \;\left[100\right]v_{t1}=\left[010\right]v_{t2}=\sqrt{C_{44}/\rho }. \end{array}\right.$$

where $v_{l}$ represents the velocity of longitudinal sound waves, $v_{t1}$ denotes the first transverse wave mode, and $v_{t2}$ corresponds to the second transverse wave mode. The calculated phase acoustic velocities for ${\mathrm{Ti}}_{3}$Si$C_{2}$, ${\mathrm{Ti}}_{3}$Ir$C_{2}$, and ${\mathrm{Ti}}_{3}$Au$C_{2}$ are presented in Table 5. Both the longitudinal and transverse sound velocities for these MAX phases decrease in the order ${\mathrm{Ti}}_{3}$Si$C_{2}$ > ${\mathrm{Ti}}_{3}$Ir$C_{2}$ > ${\mathrm{Ti}}_{3}$Au$C_{2}$. For longitudinal sound velocity, the value of [100]$v_{l}$ for each MAX phase is significantly smaller than [001]$v_{l}$, indicating the anisotropic nature of their sound velocities. As the phase acoustic velocities for each MAX phase are determined by the elastic constants, the observed anisotropy in acoustic velocities reflects the anisotropic nature of their elastic properties.

The Debye temperature, denoted as $\Theta_{D}$, serves as a pivotal property of materials, significantly influencing various physical characteristics. These include thermal conductivity, thermal expansion, lattice vibrations, specific heat capacities, and the melting point. According to Anderson’s methodology [40], $\Theta_{D}$ can be determined by employing the mean sound wave velocity ($V_{M}$) within polycrystalline materials, as follows [41]:

(17) $$\left\{\begin{array}{c} \Theta_{D}=\left(h/k_{B}\right){\left[\left(3nN_{A}\rho \right)/\left(4\pi M\right)\right]}^{1/3}V_{M},\\ \;V_{M}={\left[\left(1/3\right)\left(1/V_{L}^{3}+2/V_{T}^{3}\right)\right]}^{-1/3},\\ \;V_{L}=\sqrt{(3B_{H}+4G_{H})/3\rho },\\ \;V_{T}=\sqrt{G_{H}/\rho }. \end{array}\right.$$

Here, *h* and $k_{B}$ denote Planck’s and Boltzmann’s constants, respectively. $N_{A}$ represents Avogadro’s number, *M* is the molecular weight, *n* represents the number of atoms per molecule, and $V_{L}$ and $V_{T}$ are the longitudinal and transverse sound velocities, respectively. The calculated sound velocities $V_{L}$, $V_{T}$, $V_{M}$, and Debye temperatures $\Theta_{D}$ for ${\mathrm{Ti}}_{3}$Si$C_{2}$, ${\mathrm{Ti}}_{3}$Ir$C_{2}$, and ${\mathrm{Ti}}_{3}$Au$C_{2}$ are listed in Table 6, along with theoretical values and experimental data. The calculated values of $V_{L}$, $V_{T}$, $V_{M}$, and $\Theta_{D}$ for ${\mathrm{Ti}}_{3}$Si$C_{2}$ are consistent with previous theoretical results [7,8,9] and experimental data [5,6], confirming the accuracy and reliability of our calculations. The longitudinal ($V_{L}$), transverse ($V_{T}$), mean ($V_{M}$) velocities, and Debye temperature $\Theta_{D}$ of these MAX phases decrease in the order ${\mathrm{Ti}}_{3}$Si$C_{2}$ > ${\mathrm{Ti}}_{3}$Ir$C_{2}$ > ${\mathrm{Ti}}_{3}$Au$C_{2}$. Higher Debye temperatures are associated with better thermal conductivity in solids. Therefore, the thermal conductivities of these phases are expected to follow the same trend: ${\mathrm{Ti}}_{3}$Si$C_{2}$ > ${\mathrm{Ti}}_{3}$Ir$C_{2}$ > ${\mathrm{Ti}}_{3}$Au$C_{2}$.

Thermal conductivity is an inherent property of a material that measures its ability to transfer heat. At elevated temperatures, thermal conductivity reaches a minimal value, referred to as the minimum thermal conductivity ($K_{min}$). Clarke’s model [42] offers a method to determine this value, as follows:

(18) $$K_{min}=k_{B}V_{M}{\left(nN_{A}\rho /M\right)}^{2/3}.$$

The calculated values of Kmin for ${\mathrm{Ti}}_{3}$Si$C_{2}$, ${\mathrm{Ti}}_{3}$Ir$C_{2}$, and ${\mathrm{Ti}}_{3}$Au$C_{2}$ are listed in Table 6. Like the average sound velocities and Debye temperatures, the minimum thermal conductivities of these MAX phases are also decreased in the sequence of ${\mathrm{Ti}}_{3}$Si$C_{2}$ > ${\mathrm{Ti}}_{3}$Ir$C_{2}$ > ${\mathrm{Ti}}_{3}$Au$C_{2}$.

The melting point ($T_{m}$) of a solid is defined as the temperature at which it transitions from the solid to the liquid phase. For hexagonal crystals, $T_{m}$ can be estimated using elastic stiffness constants, as described in Ref. [43]:

(19) $$T_{m}=354+1.5\left(2C_{11}+C_{33}\right).\;$$

The calculated $T_{m}$ values for ${\mathrm{Ti}}_{3}$Si$C_{2}$, ${\mathrm{Ti}}_{3}$Ir$C_{2}$, and ${\mathrm{Ti}}_{3}$Au$C_{2}$ are presented in Table 6. The melting point exhibits a descending trend: ${\mathrm{Ti}}_{3}$Ir$C_{2}$ > ${\mathrm{Ti}}_{3}$Si$C_{2}$ > ${\mathrm{Ti}}_{3}$Au$C_{2}$, with all values exceeding 1700 K. These high melting points are promising for applications in high-temperature structural materials.

The Grüneisen parameter (γ) effectively indicates the strength of anharmonicity. A larger absolute value of γ signifies stronger phonon–phonon anharmonic dispersion, resulting in lower lattice thermal conductivity because of its inverse relationship with lattice conductivity [44]. The Grüneisen parameter can be calculated from Poisson’s ratio using the following equation:

(20) γ=1.5(1+σ)/(2−3σ).

The calculated Grüneisen parameters for ${\mathrm{Ti}}_{3}$Si$C_{2}$, ${\mathrm{Ti}}_{3}$Ir$C_{2}$, and ${\mathrm{Ti}}_{3}$Au$C_{2}$ are shown in Table 6. Among these MAX phases, ${\mathrm{Ti}}_{3}$Si$C_{2}$ has the smallest γ value, showing the weakest anharmonicity and highest lattice thermal conductivity. On the other hand, ${\mathrm{Ti}}_{3}$Au$C_{2}$ displays the strongest anharmonicity and lowest lattice thermal conductivity due to its largest γ value.

### 3.3. Density of States

**Figure 5:**
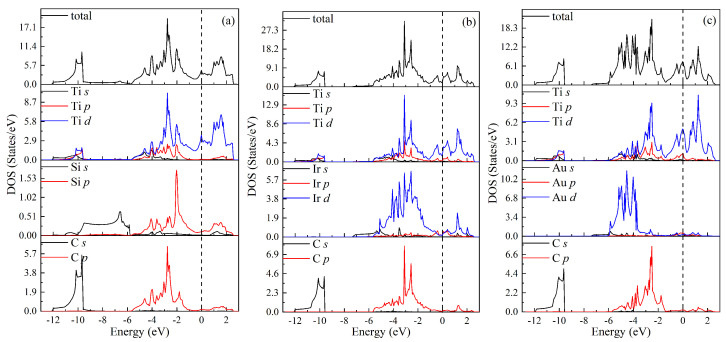
Total and partial density of states (DOS) for MAX phases: (**a**) ${\mathrm{Ti}}_{3}$Si$C_{2}$, (**b**) ${\mathrm{Ti}}_{3}$Ir$C_{2}$, and (**c**) ${\mathrm{Ti}}_{3}$Au$C_{2}$. The Fermi level is shifted to 0 eV.

**Figure 6:**
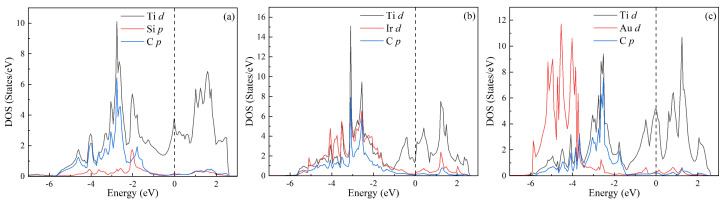
Partial density of states (DOS) for MAX phases: (**a**) ${\mathrm{Ti}}_{3}$Si$C_{2}$, (**b**) ${\mathrm{Ti}}_{3}$Ir$C_{2}$, and (**c**) ${\mathrm{Ti}}_{3}$Au$C_{2}$. The Fermi level is shifted to 0 eV.

The total and partial densities of states (DOSs) for MAX phases ${\mathrm{Ti}}_{3}$Si$C_{2}$, ${\mathrm{Ti}}_{3}$Ir$C_{2}$, and ${\mathrm{Ti}}_{3}$Au$C_{2}$ have been further calculated to understand the physical nature of the above properties, as shown in Figure 5a, Figure 5b, and Figure 5c, respectively. For each phase, the total DOS below −9 eV is determined by the *s* state of C. In the energy region between −6 eV and 0 eV, the total DOS comes mainly from Ti *d*, Si *p*, and C *p* states for ${\mathrm{Ti}}_{3}$Si$C_{2}$, from Ti *d*, Ir *d*, and C *p* states for ${\mathrm{Ti}}_{3}$Ir$C_{2}$, and from Ti *d*, Au *d*, and C *p* states for ${\mathrm{Ti}}_{3}$Au$C_{2}$. Above the Fermi level ($E_{F}$), the total DOS is attributed to Ti *d* states for ${\mathrm{Ti}}_{3}$Si$C_{2}$ and ${\mathrm{Ti}}_{3}$Au$C_{2}$, and the *d* states of Ti and Ir for ${\mathrm{Ti}}_{3}$Ir$C_{2}$. The values of total TDOS at the $E_{F}$ for ${\mathrm{Ti}}_{3}$Si$C_{2}$, ${\mathrm{Ti}}_{3}$Ir$C_{2}$, and ${\mathrm{Ti}}_{3}$Au$C_{2}$ are 4.493, 2.035, and 7.414 states/eV, respectively, indicating their metallic characteristics. Moreover, there are overlapping specifics among Ti *d*, Si *p*, and C *p* states for ${\mathrm{Ti}}_{3}$Si$C_{2}$, among Ti *d*, Ir *d*, and C *p* for ${\mathrm{Ti}}_{3}$Ir$C_{2}$, and among Ti *d*, Au *d*, and C *p* for ${\mathrm{Ti}}_{3}$Au$C_{2}$. The overlapping specifics for ${\mathrm{Ti}}_{3}$Si$C_{2}$, ${\mathrm{Ti}}_{3}$Ir$C_{2}$, and ${\mathrm{Ti}}_{3}$Au$C_{2}$ are shown in Figure 6a, Figure 6b, and Figure 6c, respectively. These imply the orbital hybridization on the formation of these phases. For each phase, the hybridization between Ti *d* and C *p* orbitals is strong. For ${\mathrm{Ti}}_{3}$Si$C_{2}$, the strong hybridizations also exist between Ti *d* and Si *p* orbitals as well as between Si *p* and C *p* orbitals. Similarly, there are the relatively strong hybridizations between Ti *d* and Ir *d* orbitals as well as Ir *d* and C *p* orbitals. However, there are weak hybridizations between Ti *d* and Au *d* orbitals as well as Au *d* and C *p* orbitals for ${\mathrm{Ti}}_{3}$Au$C_{2}$. In other words, there are directional bonding in these MAX phases, and ${\mathrm{Ti}}_{3}$Si$C_{2}$ exhibits a prominent directional feature, followed by ${\mathrm{Ti}}_{3}$Ir$C_{2}$, and ${\mathrm{Ti}}_{3}$Au$C_{2}$ exhibits a weak directional feature. These are consistent with the results of Cauchy pressures above.

## 4. Conclusions

The thermal, acoustic, mechanical, and elastic properties of ${\mathrm{Ti}}_{3}$Si$C_{2}$, ${\mathrm{Ti}}_{3}$Ir$C_{2}$, and ${\mathrm{Ti}}_{3}$Au$C_{2}$ MAX phases were comprehensively analyzed using ab initio calculations based on density functional theory. The calculated lattice parameters, along with mechanical, elastic, and acoustic properties, align well with existing experimental and theoretical findings. The mechanical stability of these MAX phases was demonstrated by their single-crystal elastic stiffness constants. From these constants, several properties were derived, including Cauchy pressures, polycrystalline elastic moduli, and directional dependencies of bulk, shear, and Young’s moduli. Additionally, various anisotropic factors were obtained from these constants. The computed Cauchy pressures, G/B ratios, and Poisson’s ratios showed that ${\mathrm{Ti}}_{3}$Au$C_{2}$ is the most ductile, followed by ${\mathrm{Ti}}_{3}$Ir$C_{2}$, with ${\mathrm{Ti}}_{3}$Si$C_{2}$ being the least ductile. Additionally, the analysis of directional elastic moduli and anisotropic factors demonstrated that incompressibility along the a and c axes follows ${\mathrm{Ti}}_{3}$Ir$C_{2}$ > ${\mathrm{Ti}}_{3}$Si$C_{2}$ > ${\mathrm{Ti}}_{3}$Au$C_{2}$. Furthermore, ${\mathrm{Ti}}_{3}$Ir$C_{2}$ and ${\mathrm{Ti}}_{3}$Au$C_{2}$ exhibit higher elastic anisotropy compared to ${\mathrm{Ti}}_{3}$Si$C_{2}$. From the single-crystal elastic stiffness constants and polycrystalline elastic moduli of these MAX phases, the pure longitudinal and transverse velocities in the principal directions, average sound velocities, Debye temperatures, minimum thermal conductivities, melting points, and Grüneisen parameters were determined. The calculated results demonstrate the following trends: ${\mathrm{Ti}}_{3}$Si$C_{2}$ exhibits higher longitudinal and transverse sound velocities, average sound velocities, Debye temperature, and minimum thermal conductivity than ${\mathrm{Ti}}_{3}$Ir$C_{2}$, which in turn exceeds ${\mathrm{Ti}}_{3}$Au$C_{2}$. The melting point follows a different trend, with ${\mathrm{Ti}}_{3}$Ir$C_{2}$ having a higher melting point than ${\mathrm{Ti}}_{3}$Si$C_{2}$, which is higher than ${\mathrm{Ti}}_{3}$Au$C_{2}$. Additionally, the Grüneisen parameter increases from ${\mathrm{Ti}}_{3}$Si$C_{2}$ to ${\mathrm{Ti}}_{3}$Ir$C_{2}$ and further to ${\mathrm{Ti}}_{3}$Au$C_{2}$.

## Figures and Tables

**Table 1:** The calculated equilibrium lattice constants (*a*, *c*, in Å), mass density (ρ, in g/${\mathrm{cm}}^{3}$), single-crystal elastic stiffness constants ($C_{ij}$, in GPa), and Cauchy pressures (${\mathrm{CP}}_{1}$, ${\mathrm{CP}}_{2}$, in GPa) of ${\mathrm{Ti}}_{3}$Si$C_{2}$, ${\mathrm{Ti}}_{3}$Ir$C_{2}$, and ${\mathrm{Ti}}_{3}$Au$C_{2}$ together with other theoretical values and experimental data.

Phase	*a*	*c*	ρ	$C_{11}$	$C_{12}$	$C_{13}$	$C_{33}$	$C_{44}$	$C_{66}$	${\mathrm{CP}}_{1}$	${\mathrm{CP}}_{2}$	Refs.
${\mathrm{Ti}}_{3}$Si$C_{2}$	3.072	17.745	4.485	363.9	85.1	98.8	351.0	155.9	139.4	−57.1	−54.4	This
Theo.	3.059	17.636	4.55	366	94	100	352	153	136	−53	−42	[7]
	3.076	17.729		360	84	101	350	158				[4,8]
	3.08	17.68		378.6	84.2	100.4	361.0	172.0				[9]
				367	86	96	351	153				[10]
	3.062	17.609		355	96	103	347	160	130	−57	−34	[11]
Expt.	3.06	17.66	4.50									[5,9]
	3.07	17.69	4.50									[6,26]
${\mathrm{Ti}}_{3}$Ir$C_{2}$	3.025	18.165	8.307	396.7	109.2	149.7	405.0	78.3	143.8	71.3	−34.6	This
	3.023	18.211										[4]
	3.025	18.196										[27]
${\mathrm{Ti}}_{3}$Au$C_{2}$	3.083	18.643	7.895	303.7	131.8	91.0	326.9	61.0	86.0	30.0	45.8	This
	3.085	18.633										[4]
	3.087	18.650										[27]
	3.084	18.657	7.87	325	116	93	324	61				[12]
Expt.		18.56										[4]

**Table 2:** The calculated bulk modulus (*B*, in GPa), shear modulus (*G*, in GPa), Young’s modulus (*E*, in GPa), and Poisson’s ratio (σ) of ${\mathrm{Ti}}_{3}$Si$C_{2}$, ${\mathrm{Ti}}_{3}$Ir$C_{2}$, and ${\mathrm{Ti}}_{3}$Au$C_{2}$ together with other theoretical values and experimental data.

Phase	$B_{V}$	$B_{R}$	$B_{H}$	$G_{V}$	$G_{H}$	$G_{H}$	$G_{H}/B_{H}$	$E_{H}$	$\sigma_{H}$	Ref.
${\mathrm{Ti}}_{3}$Si$C_{2}$	182.7	182.7	182.7	143.3	142.4	142.9	0.782	340.0	0.190	This
	186	186	186	141	140	140.5		338	0.20	[7]
			182			142				[8]
			187.6			152.9	0.82	360.7	0.18	[9]
			182			143		340	0.189	[10]
			184			140		339		[11]
Expt.	187	187	187	142	142	142	0.76	339	0.20	[5]
	185.6	185.6	185.6	143.8	143.8	143.8	0.77	343	0.192	[6]
${\mathrm{Ti}}_{3}$Ir$C_{2}$	223.9	223.2	223.6	112.7	104.5	108.6	0.486	280.5	0.291	This
${\mathrm{Ti}}_{3}$Au$C_{2}$	173.5	173.4	173.5	83.0	77.7	80.3	0.463	208.8	0.299	This
			175			87	0.495	224	0.28	[12]

**Table 3:** The calculated uniaxial bulk moduli ($B_{a}$, $B_{C}$, in GPa), and the average shear and Young’s moduli ($G_{1}$, $G_{2}$, $E_{1}$, $E_{2}$, in GPa) and Poisson’s ratios ($\sigma_{1}$, $\sigma_{2}$) on the (2$\bar{1}$0), (010), and (001) planes of ${\mathrm{Ti}}_{3}$Si$C_{2}$, ${\mathrm{Ti}}_{3}$Ir$C_{2}$, and ${\mathrm{Ti}}_{3}$Au$C_{2}$.

Phase	$B_{a}$	$B_{c}$	$G_{1}$	$G_{2}$	$E_{1}$	$E_{2}$	$\sigma_{1}$	$\sigma_{2}$	Ref.
${\mathrm{Ti}}_{3}$Si$C_{2}$	547.5	549.2	147.2	155.9	326.4	307.5	0.202	0.220	This
					311	300	0.201	0.229	[11]
${\mathrm{Ti}}_{3}$Ir$C_{2}$	627.0	774.9	101.4	78.3	332.9	316.4	0.235	0.296	This
${\mathrm{Ti}}_{3}$Au$C_{2}$	533.2	496.1	71.4	61.0	237.7	288.9	0.277	0.209	This

**Table 4:** The calculated anisotropic factors of ${\mathrm{Ti}}_{3}$Si$C_{2}$, ${\mathrm{Ti}}_{3}$Ir$C_{2}$, and ${\mathrm{Ti}}_{3}$Au$C_{2}$.

Phase	$A_{1}$	$A_{2}$	$A_{3}$	$A^{U}$	$A^{L}$
${\mathrm{Ti}}_{3}$Si$C_{2}$	1.205	1.205	1	0.032	0.014
${\mathrm{Ti}}_{3}$Ir$C_{2}$	0.624	0.624	1	0.397	0.169
${\mathrm{Ti}}_{3}$Au$C_{2}$	0.544	0.544	1	0.337	0.146

**Table 5:** The calculated longitudinal and transvers sound velocities (m/s) of ${\mathrm{Ti}}_{3}$Si$C_{2}$, ${\mathrm{Ti}}_{3}$Ir$C_{2}$, and ${\mathrm{Ti}}_{3}$Au$C_{2}$.

Phase	$\left[100\right]v_{l}$	$\left[010\right]v_{t1}$	$\left[001\right]v_{t2}$	$\left[001\right]v_{l}$	$\left[100\right]v_{t1}$	$\left[010\right]v_{t2}$
${\mathrm{Ti}}_{3}$Si$C_{2}$	1763	2849	1865	2798	1865	1865
${\mathrm{Ti}}_{3}$Ir$C_{2}$	1316	2185	971	2208	971	971
${\mathrm{Ti}}_{3}$Au$C_{2}$	1044	1961	879	2035	879	879

**Table 6:** The calculated elastic wave velocities ($V_{L}$, $V_{T}$, $V_{M}$, in m/s), Debye temperatures ($\Theta_{D}$, in K), melting points ($T_{m}$, in K), minimum thermal conductivities ($K_{min}$, in W/mK), and Grüneisen parameter (γ) of ${\mathrm{Ti}}_{3}$Si$C_{2}$, ${\mathrm{Ti}}_{3}$Ir$C_{2}$, and ${\mathrm{Ti}}_{3}$Au$C_{2}$.

Phase	$V_{L}$	$V_{T}$	$V_{M}$	$\Theta_{D}$	$K_{\min}$	$T_{m}$	γ	Ref.
${\mathrm{Ti}}_{3}$Si$C_{2}$	9122	5644	6224	807.4	1.635	1972.3	1.248	This
	9069	5571	6148	801				[7]
				805				[8]
	9330	5830		834.3				[9]
Expt.	9142	5613	6195	784				[5]
	9185	5670	6225	813				[6]
${\mathrm{Ti}}_{3}$Ir$C_{2}$	6659	3616	4035	524.7	1.083	2151.6	1.718	This
${\mathrm{Ti}}_{3}$Au$C_{2}$	5962	3190	3563	453.6	0.914	1755.4	1.769	This

## Data Availability

The original contributions presented in this study are included in the article. Further inquiries can be directed to the corresponding author.
